# Effect of light intensity on celery growth and flavonoid synthesis

**DOI:** 10.3389/fpls.2023.1326218

**Published:** 2024-01-16

**Authors:** Yanmei Qin, Xuanxuan Liu, Chunyan Li, Qianwen Chu, Shaobo Cheng, Lihong Su, Dalong Shao, Xin Guo, Zhongqun He, Xiaoting Zhou

**Affiliations:** College of Horticulture, Sichuan Agricultural University, Chengdu, China

**Keywords:** light intensity, celery, nutrients, quality, flavonoid

## Abstract

Light is one of the important environmental factors affecting the growth and development of facility vegetables. In this experiment, we investigated the effects of different light intensities on the growth, nutritional quality and flavonoid accumulation of celery under hydroponic and full LED light conditions. Four light intensities of 40, 100, 200, or 300 µmol·m^-2^·s^-1^ were set up in the experiment, and three harvest periods were set up on the basis of different light intensities, which were 15, 30, and 45 d after treatment (labeled as S1, S2, and S3, respectively). The results showed that the plant height and aboveground biomass of celery increased with the increase of light intensity, and the light intensity of 200 μmol·m^-2^·s^-1^ was beneficial to increase the contents of chlorophyll, carotenoids, total phenols, vitamin C, cellulose, total flavones and apigenin in celery. During the S1-S3 period, the activities of PAL, CHS, CHI and ANS increased gradually under 200 and 300 μmol·m^-2^·s^-1^ light intensity treatments, and the activities of FNS and CHS enzymes were the highest under 200 μmol·m^-2^·s^-1^ light intensity treatment. The expression and ANS activity of *Ag3GT*, a key gene for anthocyanin synthesis, reached the maximum value at 300 μmol·m^-2^·s^-1^, and the expression level and FNS activity of *AgFNS*, a key gene for apigenin synthesis, reached a maximum value at 200 μmol·m^-2^·s^-1^. In general, the anthocyanin content was the highest at 300 μmol·m^-2^·s^-1^, and the apigenin content was the highest at 200 μmol·m^-2^·s^-1^. In conclusion, light intensity of 200 µmol·m^-2^·s^-1^ treatment was more favorable for celery growth and nutrient synthesis.

## 1 Introduction

Light is an important source of energy and signaling for plants, which influences plant growth, development, and structural characteristics. The morphological structure and material accumulation of plants are closely related to light intensity, and both low and high light are detrimental to plant growth (Lv et al., 2021). When plants are exposed to low-light environments for long periods of time, they tend to develop a series of shade-tolerant mechanisms in order to adapt to such environments, such as increasing plant height to obtain more light energy, decreasing leaf area, decreasing energy consumption and transpiration, promoting plant stem elongation, decreasing root biomass, and enhancing apical dominance (Aphalo and Ballaré, 1995; Ballaré and Scopel, 1997; Collins and Wein, 2000). When the light intensity is too strong, the tissue structure of plant leaves will change, the leaves will become smaller and thicker, and the light intensity is too strong to affect the transportation and transfer of nutrients and water in the plant body, and the leaves will turn yellow or even wither (Matos et al., 2009). Studies have shown that the content of vitamin C and soluble sugars decreases when light intensity is insufficient, suggesting that low light has an inhibitory effect on the synthesis of vitamin C and soluble sugars (Fu et al., 2017; Liu et al., 2022). In addition, with the increase of light intensity, the content of ascorbic acid in lettuce showed an increasing trend, but the content of soluble protein and nitrate in lettuce showed a decreasing trend (Fu et al., 2017). In conclusion, too much or too little light can inhibit plant growth, so only at the appropriate light intensity can we better promote the quality formation of vegetables.

Flavonoids are important secondary metabolites in plants with pharmacological effects such as anticancer, antioxidant and hypoglycemic. They generally refer to a series of compounds formed by two benzene rings interconnected by a central three-carbon chain (Bast et al., 2007; Mojžiš et al., 2008). Flavonoids can be categorized into eight types according to their substituents: flavonoids, flavonols, dihydroflavonoids, chalcones, isoflavonoids, dioflavonoids, anthocyanins and flavanols (Safe et al., 2021). Anthocyanins and apigenin are important secondary metabolites in plants and belong to flavonoids. For plants, light is a crucial environmental factor affecting the synthesis of flavonoids, which plays a role in two ways: Light not only affects plant morphogenesis and growth metabolism, provides the necessary metabolic substrates for the synthesis of flavonoids, but also promotes the synthesis of flavonoids by activating enzymes that regulate flavonoid metabolism. Studies have shown that different light intensities, light qualities and photoperiods can indirectly regulate the expression of flavonoid-regulated genes and thus control the accumulation of flavonoids through the modulation of photosensitive pigments and cryptochromes (Jaakola, 2013). Benjamín Battistoni et al. demonstrated that different ratios of light quality composition favored the synthesis of spinach flavonoids (Samuolienė et al., 2012). The changes in flavonoid content in kale leaves revealed that the accumulation of quercetin glucoside analog synthesis was promoted under strong light conditions, whereas the accumulation of kaempferol glucoside analog synthesis was inhibited, but the total flavonoid content was consistently higher in strong light than in weak light (Neugart et al., 2016).

Celery (*Apium graveolens* L.) is a widely cultivated vegetable in the world with rich nutritional and health values. There is the efficacy of lowering blood pressure, strong antioxidant and free radical scavenging ability, cancer prevention and anti-cancer in celery. Especially, anthocyanins and apigenin are important flavonoids in celery and have the ability to improve the body’s defense against diseases. In addition to varietal differences, its components as well as content are also affected by environmental factors such as light, but there are fewer studies on the dynamics of flavonoid compounds in celery, as well as the correlation between the corresponding physiological indexes and light intensity.

In this study, Hongcheng red celery (HQ) was used as the test material to determine the effects of light intensity on the growth, basic nutrients and the content of important flavonoids in the body of celery under the conditions of plant factories. By exploring the effects of light intensity on the synthesis and accumulation of flavonoid compounds in different developmental stages of celery leaves and petioles, and then understanding the changes in the synthesis and accumulation of compounds under the treatment of different light intensities, we can provide a reference for the molecular mechanism of light regulation of the synthesis and accumulation of flavonoid compounds in celery. At the same time, it can also provide scientific basis for the promotion and cultivation management of celery in production, and provide reference value for the reasonable control of light in practice.

## 2 Materials and methods

### 2.1 Materials and seedlings cultivation

Experiments were conducted in a plant factory at Sichuan Agricultural University (Chengdu, China) in 2021. The selected material was Hongcheng red celery (*Apium graveolens* L.) Full and healthy celery seeds were soaked in warm broth and then germinated in a climatic chamber, where the temperatures were maintained at 18°C ± 2°C (day) and 15°C ± 2°C (night). After the seeds were dewy, they were sown in black cross seedling sponges (2.5 × 2.5 × 2.5 cm) with round holes and two seeds were sown in each hole. When the seedlings reached two leaves and one heart, they were watered with 1/2 of Hoagland’s nutrient solution. The pH of the nutrient solution was 6.5 ± 0.5 and the EC was 1.2 ± 0.5 ms·cm^-1^.

### 2.2 Experimental treatment

When the seedlings are five leaves and one heart (about 60 d after sowing), they are selected and moved into the hydroponic cycle racks of the plant factory and kept at 1 plant per hole. These seedlings were shosen in uniform growth, with free of pests and diseases. The light intensity of the light source was adjusted with a plant light analyzer. The experiment was set up with four treatments, 40 µmol·m^-2^·s^-1^, 100 µmol·m^-2^·s^-1^, 200 µmol·m^-2^·s^-1^, and 300 µmol·m^-2^·s^-1^. Each treatment had 35 seedlings and was replicated three times for a total of 105 seedlings. The environmental conditions were maintained at a temperature of 25°C/18°C (day/night), air humidity of 50%, and a photoperiod of 12/12 h (day/night) (Pre-experimental screening). After transplanting, the seedlings were watered with 1/2 Hoagland nutrient solution at the same pH as that of seedlings and an EC value of 2.0 ± 0.5 ms·cm^-1^. The content of flavonoids, flavonoid-related enzyme activities, and expression of related genes in celery were determined at 15, 30, and 45 d after treatment (S1, S2, and S3, respectively).

### 2.3 Determination of growth parameters

After 45 d of treatment with different light intensities, we harvested celery. Plant height and root length were measured with a millimeter scale. Aboveground dry and fresh weights were measured with an electronic balance, and water content was calculated using the formula: (fresh weight - dry weight)/fresh weight × 100%.

### 2.4 Assay for chlorophyll content

After 45 d of treatment with different light intensities, we harvested celery. For each treatment, 0.5 g of fresh leaves were weighed and the photosynthetic pigment content of celery leaves was determined with reference to the acetone-ethanol mixture immersion method (Lichtenthaler and Buschmann, 2001). The chlorophyll content was calculated as follows:


$$\text{Chl a }\left(\text{mg}/\text{g}\right)=\frac{\left(12.72\times \text{OD}663-2.59\times \text{OD}645\right)\text{ V}}{1000\text{W}}$$

$$\text{Chl b}\left(\text{mg}/\text{g}\right)=\frac{\left(22.88\times \text{OD}645-4.67\times \text{OD}663\right)\text{ V}}{1000\text{W}}$$

$$\text{Car}(\text{mg}/\text{g})=\frac{\left(1000\;\times \text{ OD}470\;-\;3.27\;\times \text{ Chl}.\text{a }-\;104\;\times \text{ Chl}.\text{b}\right)/229)\text{ V}}{1000\text{W}}$$

### 2.5 Measurement of chlorophyll fluorescence and photosynthetic parameters

After 45 d of treatment with different light intensities, five fully expanded mature leaves from each treatment were randomly selected and the maximum photosynthetic efficiency (Fv/Fm), photochemical burst (qP), non-photochemical burst (NPQ), and relative electron transfer rate (ETR) of photosystem II were determined by a portable fluorometer (Walz, PAM-2500, Effeltrich, Germany). Net photosynthetic rate (Pn), intercellular CO_2_ concentration (Ci) stomatal conductance (Gs), and transpiration rate (Tr) of leaves were determined with a portable photosynthesis meter (Li-6400,United State).

### 2.6 Measurement of essential nutrients

After 45 d of treatment with different light intensities, we divided the celery into leaves and petioles and took 0.5 g of fresh samples for the determination of the basic nutrients they contained. Nitrate content in celery leaves was measured with reference to the salicylic acid-sulfuric acid colorimetric method (Cataldo et al., 1975). Vitamin C content was determined by molybdenum blue spectrophotometry (Chen et al., 2015). The total phenolic content in methanolic extracts was determined using the Folin-Ciocalteau procedure with gallic acid as a standard (Singleton et al., 1999). Cellulose content was measured by digestion and gravimetric technique (Antia et al., 2006).

### 2.7 Measurement of major flavonoid compounds content

The content of major flavonoid compounds contained in celery was determined at 15, 30 and 45 d after treatment with different light intensities. The extract was carried out with methanol at a material ratio of 1:25, and the total flavonoid content was determined after ultrasonication and filtration (Ghadage et al., 2017). The anthocyanin content was determined by the method of Li (Li et al., 2012). According to: OD = (OD530 - OD620) - 0.1 × (OD650 – OD620) formula, the anthocyanin absorbance value in each sample was calculated, and the anthocyanin content in each sample was calculated according to the total anthocyanin content per 1 mg sample per 0.1 absorbance. Apigenin content was determined by liquid chromatography (Kim et al., 2008; Yan et al., 2014).

### 2.8 Measurement of flavonoid-related enzyme activities

The activities of flavonoid-related enzymes in celery were measured at 15, 30 and 45 days after different light intensities. The activities of phenylalanine deaminase (PAL), chalcone isomerase (CHI), chalcone synthase (CHS), flavonoid synthase (FNS) and anthocyanin synthase (ANS) during the synthesis of flavonoids were measured using ELISA kits produced by Chongqing Bonoheng Biotechnology Co.

### 2.9 Expression analysis of key flavonoid-related genes

*Ag3GT*, a key gene for anthocyanin synthesis, and *AgFNS*, a key gene for apigenin synthesis, were identified quantitatively (Tan, 2020). The expression levels of apigenin and anthocyanin-related genes were measured at 15, 30 and 45 days after different light intensities. Total RNA was extracted from the leaves and petioles of fresh celery by employing an RNA preparation kit (Tiangen, Beijing; 0.1 g). 1µg of total RNA was reverse transcribed to cDNA using TaKaRa reverse transcription kit, then diluted with sterile ddH_2_O and stored in -20°C refrigerator and used for fluorescent quantitative PCR analysis. The fluorescent dye reagent is TB GreenTM Permix Ex TapTM II (TAKARA). Use the Archimed X Series real-time PCR instruments. The amplification program was as follows: 30 s at 95°C, 40 cycles of 5 s at 95°C, 15 s at 58°C. Relative gene expression was evaluated using the 2^-△△CT^ method. The PCR primers were designed using Primer Premier 5 software (PREMIER Biosoft, Palo Alto, CA, United States), and is listed in **Table 1**. Actin (AF111812) was used as an internal control.

**Table 1:** Primers for expression of anthocyanin and apigenin metabolism in celery.

Gene	Forward primers (5’-3’)	Reverse primers (5’-3’)
*AgACTIN*	CTTCCTGCCATATATGATTGG	GCCAGCACCTCGATCTTCATG
*Ag3GT*	TGTTTGGGAACTTAGAGTCTCCTT	TATTGTAATGCTGGTGGTGATGTA
*AgFNS*	AAGGCGGCTTTACTATCTCCACTC	CACCTAGCACCATTAACTTCTCAC

### 2.11 Statistical analysis

Trials were statistically and graphically plotted using Origin 2017, and SPSS 27.0 software was used to perform analysis of significant variance (ANOVA) on the data, and Duncan’s multiple test was used to compare the means.

## 3 Results and analysis

### 3.1 Effect of light intensity on biological parameters of celery

**Figure 1:**
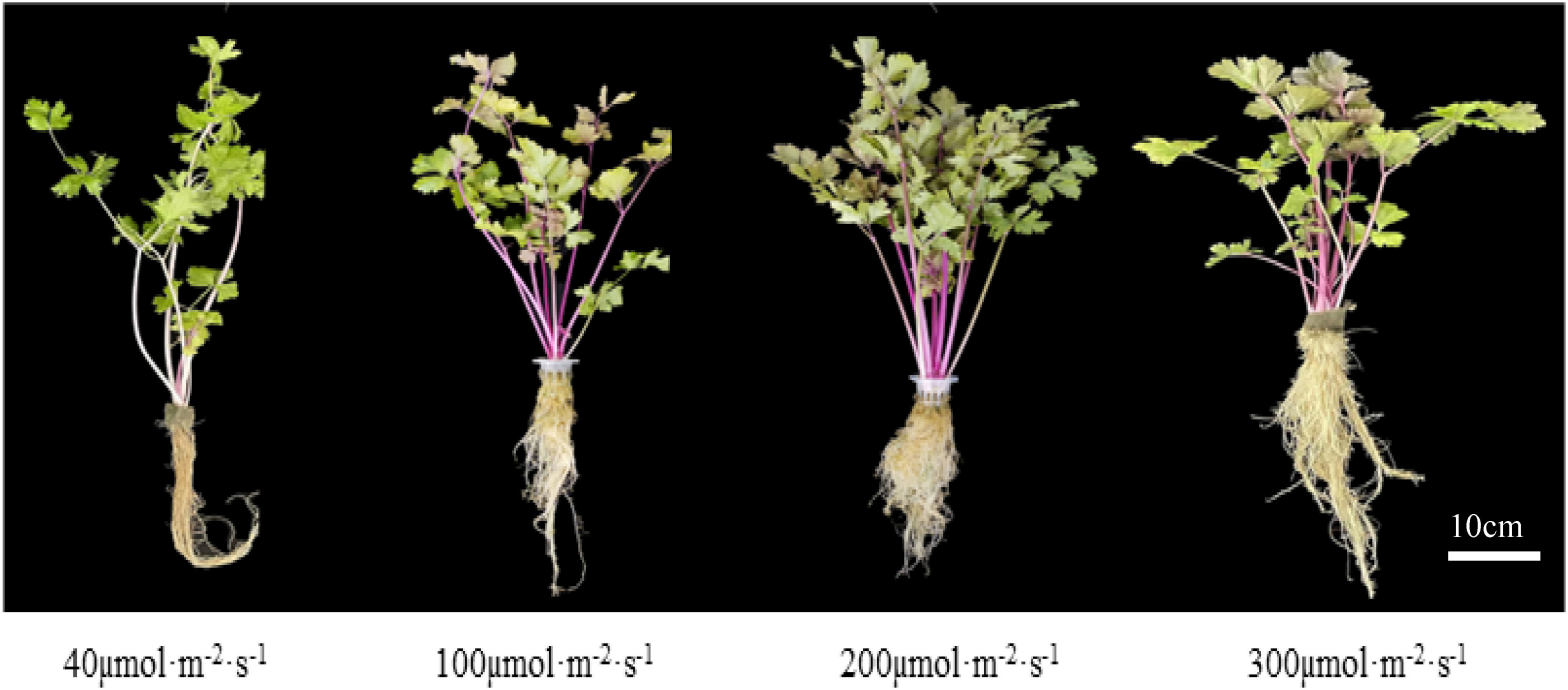
Celery plants grown under four different light intensities 45 days after treatment.

The effects of different light intensities on the changes of various morphological indicators of celery were basically the same. With increasing light intensity, celery height, fresh weight and dry weight tended to increase and then decrease, while root length decreased (**Figure 1**). The above-ground fresh and dry weights of celery increased by 64.07% and 205.17%, respectively, when the light intensity was 200 µmol·m^-2^·s^-1^ compared with that of 40 µmol·m^-2^·s^-1^. This indicates that suitable light intensity can promote the growth of celery (**Table 2**).

**Table 2:** Effects of different light intensities on celery biomass.

Treatment μmol·m^-2^·s^-1^	Plant height (cm)	Root length (cm)	Shoot fresh weight (g)	Shoot dry weight (g)	Shoot water content (%)
40	40.37 ± 3.742b	40.03 ± 4.325a	16.70 ± 1.111c	0.58 ± 0.088c	96.53 ± 0.276a
100	39.17 ± 0.305b	26.80 ± 4.073b	24.78 ± 0.865b	0.98 ± 0.458b	96.03 ± 0.261b
200	47.23 ± 2.400a	20.04 ± 5.481d	27.40 ± 1.043a	1.77 ± 0.399a	93.55 ± 0.354d
300	28.03 ± 0.451c	21.60 ± 5.161c	25.03 ± 0.895b	1.11 ± 0.187b	95.58 ± 0.375c

Different lowercase letters indicate significant differences (p< 0.05) within the same column of the table.

The photosynthetic pigment content of celery leaves increased and then decreased with increasing light intensity, and the contents of chlorophyll a, chlorophyll b, total chlorophyll and carotenoids were highest at a light intensity of 100 µmol·m^-2^·s^-1^and lowest at a light intensity of 300 µmol·m^-2^·s^-1^ for all photosynthetic pigments except carotenoids (**Table 3**).

**Table 3:** Effects of different light intensities on photosynthetic pigment content of celery.

Treatment μmol·m^-2^·s^-1^	Chlorophyll a (mg/g)	Chlorophyll b (mg/g)	Total chlorophyll (mg/g)	Carotenoids (mg/g)
40	0.444 ± 0.039b	0.206 ± 0.011a	0.650 ± 0.047b	0.022 ± 0.001d
100	0.477 ± 0.036a	0.208 ± 0.013a	0.685 ± 0.050a	0.070 ± 0.001a
200	0.317 ± 0.028c	0.140 ± 0.010b	0.457 ± 0.037c	0.061 ± 0.003b
300	0.232 ± 0.023d	0.104 ± 0.013c	0.336 ± 0.057d	0.049 ± 0.002c

Different lowercase letters indicate significant differences (p< 0.05) within the same column of the table.

### 3.2 Effect of light intensity on Photosynthetic characteristics in celery

**Figure 2:**
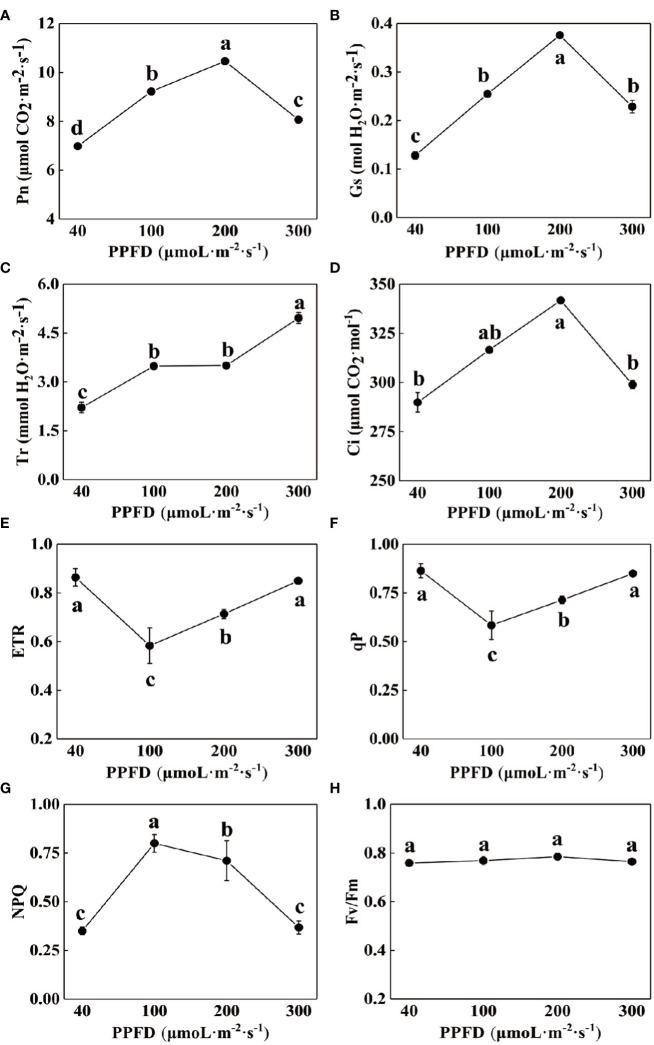
Effects of different light intensities on photosynthetic gas exchange parameters and chlorophyll fluorescence parameters of celery. **(A)** net photosynthetic rate (Pn), **(B)** stomatal conductance (Gs), **(C)** transpiration rate (Tr), **(D)** intercellular CO2 concentration (Ci), **(E)** Electron transport rate through PSII (ETR), **(F)** photochemical quenching coefficient (qP), **(G)** Non-photochemical quenching (NPQ) and **(H)** The maximum PSII quantum yield (Fv/Fm) of celery leaves in response to 40, 100, 200 and 300 µmol·m^-2^·s^-1^ photosynthetic photon flux density (PPFD). The mean ± SE are shown (n ≥ 3). Different letters indicate significant difference among the treatments (P< 0.05).

Different light intensities affected the photosynthetic capacity of celery. The values of Pn, Gs and Ci all varied in an ascending and then descending manner, reaching a maximum at a light intensity of 200 µmol·m^-2^·s^-1^(**Figures 2A, B, D**). The value of Tr reached a maximum at a light intensity of 300 µmol·m^-2^·s^-1^, which was higher than that at a light intensity of 40 µmol·m^-2^·s^-1^ 124.43% (**Figure 2C**).

Chlorophyll fluorescence parameters reflect the process of absorption, transmission and dissipation of light energy by the photosystem and are often used to test the photosynthetic capacity of plants. The NPQ of celery leaves increased and then decreased with increasing light intensity, and was significantly higher than the other treatments at a light intensity of 100 µmol·m^-2^·s^-1^, 128.57%, 12.68%, and 116.22% (**Figure 2G**), respectively, while ETR and qP decreased and then increased (**Figures 2E, F**), and Fv/Fm did not change significantly (**Figure 2H**).

### 3.3 Effect of light intensity on essential nutrients in celery

**Figure 3:**
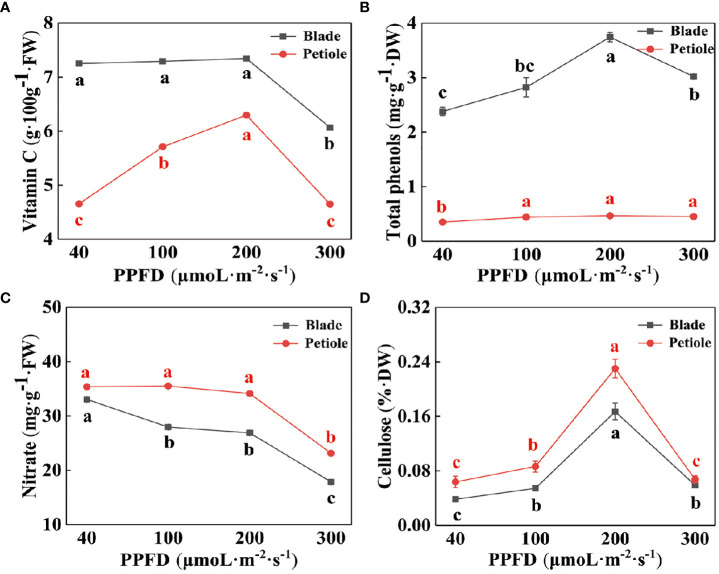
The contents of vitamin C **(A)**, total phenolics **(B)**, nitrate **(C)** and cellulose **(D)** in celery leaves and petioles under different photosynthetic photon flux density (PPFD). Different letters (a–d) on the bar plots indicate significant difference at p< 0.05 using one-way analysis of variance with Duncan’s multiple-range test.

**Figure 3** reflects the changes in essential nutrients in celery leaves under different light intensity treatments. The vitamin C and total phenolics contents of celery leaves were higher than those of petioles (**Figures 3A, B**), and the cellulose and were lower than those of petioles (**Figure 3D**). All the indicators except nitrate showed a trend of increasing and then decreasing with increasing light intensity, reaching a maximum at a light intensity of 200 µmol·m^-2^·s^-1^. The content of nitrate decreased with increasing light intensity and was significantly lower at a light intensity of 300 µmol·m^-2^·s^-1^ (**Figure 3C**). Compared with a light intensity of 40 µmol·m^-2^·s^-1^, the nitrate content of leaves and petioles respectively decreased by 33.88% and 38.96%.

### 3.4 Effect of light intensity on important flavonoid compounds

**Figure 4:**
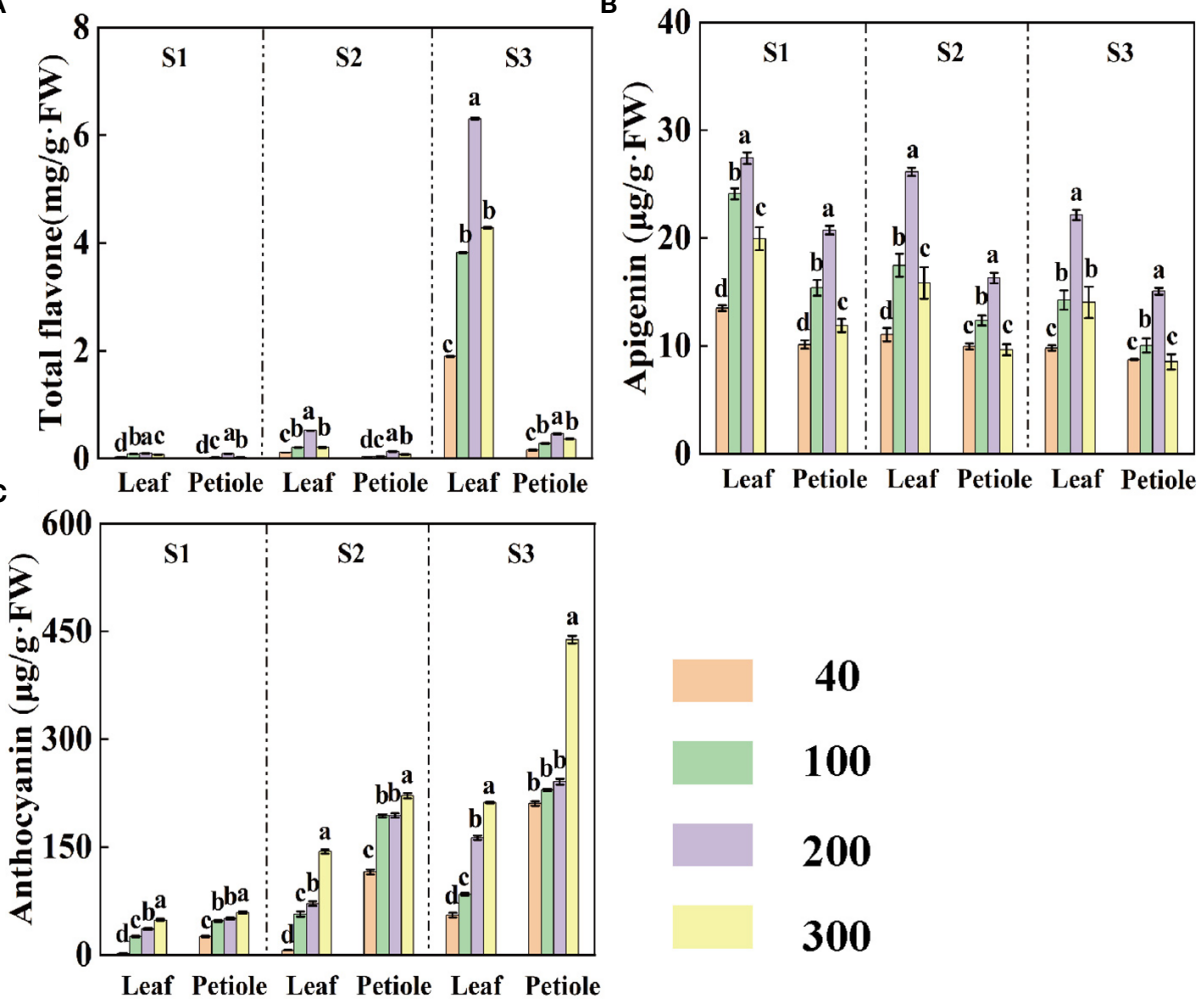
The contents of total flavones **(A)**, apigenin **(B)** and anthocyanins **(C)** in celery at three different periods (S1 15d, S2 30d, S3 45d) under different light intensity treatments. Different letters (a–c) on the bar plots indicate significant difference at p< 0.05 using one-way analysis of variance with Duncan’s multiple-range test.

Suitable light is beneficial in promoting the synthesis of important flavonoids in celery. Celery petioles have higher levels of anthocyanins than leaves (**Figure 4C**), while leaves contain higher levels of apigenin than petioles (**Figure 4B**). During the same period, the content of total flavones and apigenin increased and then decreased with increasing light intensity, reaching a maximum at a light intensity of 200 µmol·m^-2^·s^-1^ (**Figures 4A, B**), while the content of anthocyanins continued to be enhanced (**Figure 4C**). The anthocyanin content of leaves and petioles gradually increased with increasing treatment time, but apigenin showed the opposite trend (**Figure 4**). In S3 period, the anthocyanin content of leaves and petioles was 344.48%, 644.44% and 47.43%, 98.13% higher at light intensity of 300 µmol·m^-2^·s^-1^ compared to S1 and S2 periods. The apigenin content was the highest at 200 μmol·m^-2^·s^-1^ in celery, and the apigenin content in leaves and petioles at S3 period decreased by 22.47 %, 37.62 %, 18.01 % and 8.23 % compared with S1 and S2 periods (**Figure 4**).

### 3.5 Effect of light intensity on PAL, CHI, CHS, FNS and ANS

**Figure 5:**
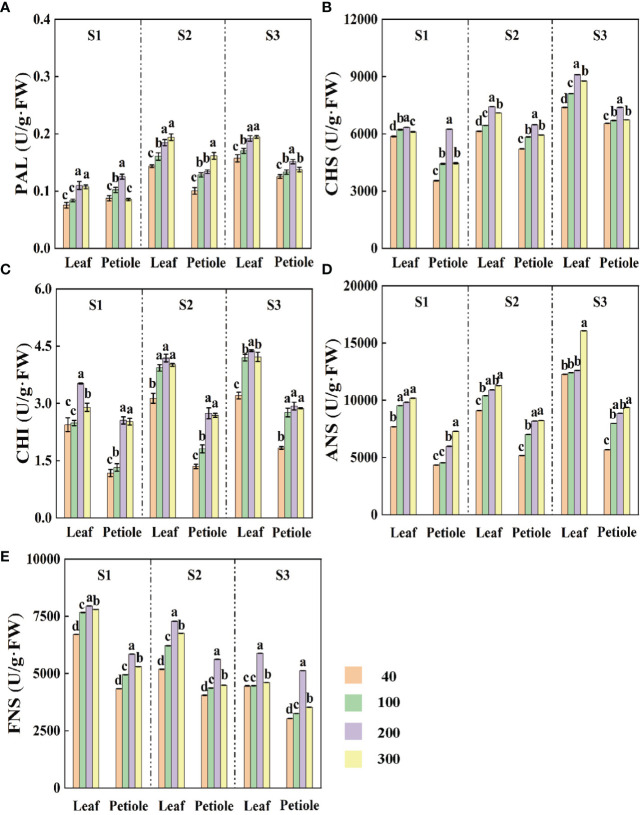
The effects of light intensity on **(A)** phenylalanine deaminase PAL, **(B)** chalcone synthase CHS, **(C)** chalcone isomerase CHI, **(D)** anthocyanin synthase ANS, and **(E)** flavonoid synthase FNS at three different periods (S1 15d, S2 30d, S3 45d). Different letters (a–e) on the bar plots indicate significant difference at p< 0.05 using one-way analysis of variance with Duncan’s multiple-range test.

Except for the ANS activity which increased with the increase of light intensity, the activities of all the enzymes showed a trend of increasing and then decreasing with the increase of light intensity, and reached the maximum at the light intensity of 200 µmol·m^-2^·s^-1^ (**Figure 5**). Under the same light intensity treatment, the activities of PAL, CHS, CHI and ANS were enhanced with increasing treatment time (**Figures 5A–D**), but the activity of FNS was gradually decreased (**Figure 5E**). The lowest FNS activity decreased by 56.01%, 43.01% and 14.14% and 9.59% in S3 period compared with S1 and S2, respectively (**Figure 5E**).

### 3.6 Effect of light intensity on apigenin and anthocyanin gene expression

**Figure 6:**
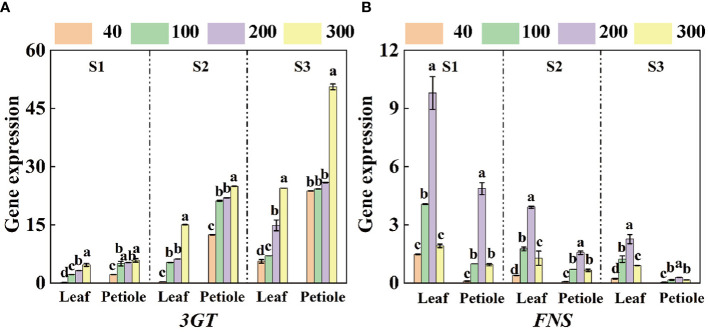
qRT-PCR analyzed the expression of genes related to apigenin and anthocyanin synthesis in celery blades and petioles. **(A, B)** The expression levels of 3GT and FNS in three different periods (S1 15d, S2 30d, S3 45d). The mean ± SE are shown (n ≥ 3). Different letters indicate significant difference among the treatments (P< 0.05).

The expression of anthocyanin synthesis gene *Ag3GT* increased with the increase of light intensity, while the expression of apigenin synthesis gene *AgFNS* showed a trend of increasing and then decreasing, and reached the maximum at the light intensity of 200 µmol·m^-2^·s^-1^ (**Figure 6**). The expression of *AgFNS* showed a decreasing trend under the same light intensity treatment and the expression of *AgFNS* in the leaves was significantly higher than that in the petioles (**Figure 6B**). The expression of *Ag3GT* increased with the increase of treatment time under the same light intensity treatment, and the maximum expression of the gene in the leaf and petiole increased 422.86%, 760.20% and 62.59%, 102.32% in S3 period compared with S1 and S2 periods, respectively, and the maximum expression in the petiole was 2.07 times higher than that in the leaf (**Figure 6A**).

### 3.7 Correlation analysis of light intensity on anthocyanin and apigenin content of celery

**Figure 7:**
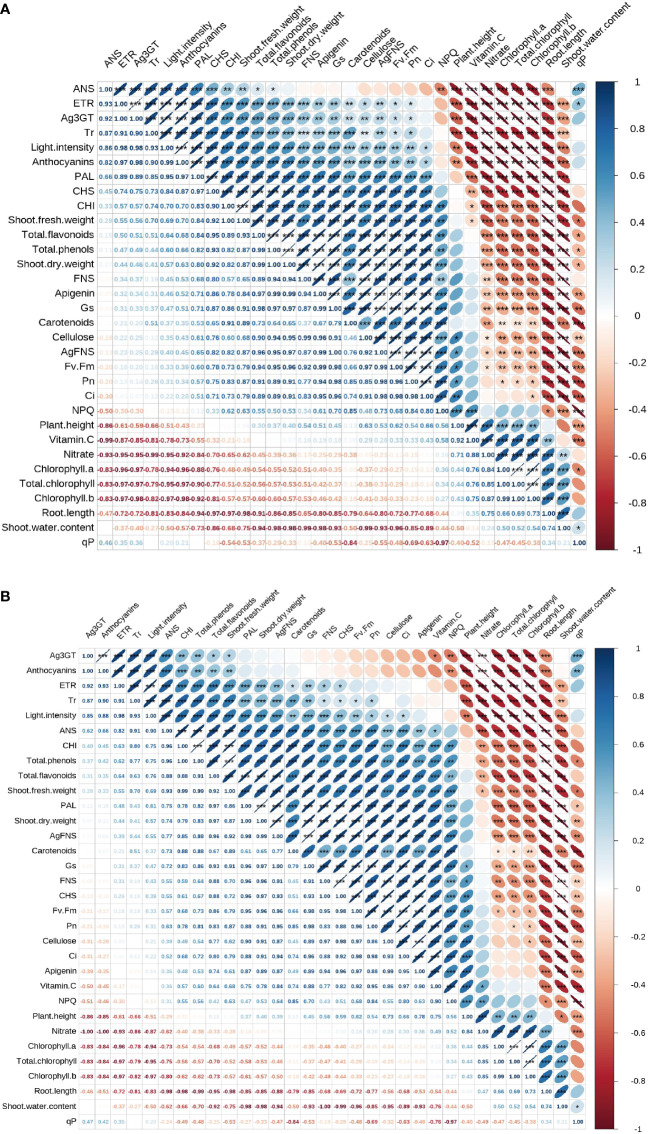
Correlation analysis of light intensity on anthocyanin and apigenin content. Correlation analysis of light intensity on anthocyanin and apigenin content of celery blade **(A)** and petioles **(B)**.

**Figure 7** shows the correlation analysis of light intensity on apigenin and apigenin content. In celery blades (**Figure 7A**), the light intensity was significantly correlated with the anthocyanin content and apigenin content of celery blades. The content of apigenin in celery blades was significantly negatively correlated with the shoot water content of celery, and was positively correlated with the expression of stem thickness, aerial dry weight, Gs, Ci, Fv/Fm, blade total phenols, *AgFNS*, total flavonoids, PAL, CHS, FNS and *AgFNS.* The anthocyanin content was positively correlated with PAL activity and *Ag3GT* expression in blades, but negatively correlated with chlorophyll a, chlorophyll b and total chlorophyll content in blades. In celery petioles, the light intensity was significantly negatively correlated with the total chlorophyll content in celery petioles, and its ETR was significantly positively correlated.

In celery petioles (**Figure 7B**), light intensity was significantly negatively correlated with the total chlorophyll content in celery petioles. The content of apigenin in the petiole was positively correlated with CHS activity and cellulose content. The anthocyanin content in the petiole was significantly negatively correlated with the total chlorophyll content, and the nitrate content and *Ag3GT* expression in the petiole were significantly positively correlated.

## 4 Discussion

Light is one of the most influential environmental factors on plants, and light intensity plays a crucial role in the morphological construction and material accumulation of plants. The fresh weight of Brassica microgreens increased 34% as light intensity increased from 105 to 315 μmol·m^-2^·s^-1^ (Gerovac et al., 2016). The length and fresh weight of tartary buckwheat sprouts can be affected by light, even if the intensity is very low (50 μmol·m^-2^·s^-1^) (Tuan et al., 2013). The 50 μmol·m^-2^·s^-1^ treatment increased fresh weight and hypocotyl length of broccoli microgreens (Liu, 2021). In this study, the aboveground biomass and water content of celery were significantly reduced under low light conditions, and the height and aboveground biomass of celery plants were highest and root development was most vigorous at a light intensity of 200, while the accumulation of each biomass of celery was also significantly inhibited at a light intensity of 300 (**Table 1**).

Zhang found that too much or too little light treatment reduced the photosynthetic pigment content in plant leaves and inhibited photosynthesis in plants (Zhang et al., 2021). Chll a, Chll b and Chll a + b contents in celery leave were lowest at light intensity of 40, and chlorophyll content at light intensity of 300 was lower than that at light intensity of 200, indicating that chlorophyll in celery leave is less tolerant to both low light environment and high light intensity, and the decrease in chlorophyll content under strong light may be caused by damage or reduction in the number of basal grains in chloroplasts (**Table 2**). The appropriate light intensity can reduce the damage to the leave and the formation of related tissue structures, which in turn is conducive to promoting chlorophyll synthesis (Feng et al., 2019; Wang T. et al., 2022). The present results indicates that the transpiration rate (Tr) and net photosynthetic rate (Pn) of grass coral gradually increased to a certain level and then decreased with the increase of light intensity in a certain range of light intensity (Shi-Jian et al., 2015). In our study, we found that Pn, GS and Ci of celery all showed an increasing and then decreasing trend in the light intensity range of this experiment (**Figures 2A, B, D**), while Tr gradually increased with the increase of light intensity, which might be due to the differences caused by different plant species (**Figure 2C**), indicating that the suitable light intensity is beneficial to promote photosynthesis of celery.

Chang found that the nitrate content in leaf lettuce was reduced by increasing the light intensity appropriately, and the results of the present experiment were consistent with this (Chang et al., 2013). With increasing light intensity, nitrate content decreases and decreases significantly at a light intensity of 300 μmol·m^-2^·s^-1^ (**Figure 3C**). Studies have shown that the content of soluble sugar, cellulose, and VC in water spinach increases significantly with increasing light intensity (Glowacz et al., 2015). However, In this study, the contents of VC and cellulose in celery petioles and leaves increased first and then decreased with the increase of light intensity, and reached a maximum when the light intensity was 200 μmol·m^-2^·s^-1^ (**Figures 3A, D**). This may be due to different experimental settings or differences between plant species. Increasing light intensity is beneficial to the synthesis of soluble proteins and total phenols, but too much light intensity will play an inhibitory role (Miao et al., 2023). In this study, the total phenolic content decreased significantly when the light intensity increased to 300 µmol·m^-2^·s^-1^, which may be due to the fact that the synthesis process of phenolic compounds is regulated by phenylalanine via phenylalanine aminolysin (PAL), while high light intensity affected PAL activity (**Figure 3B**).

Studies have shown that the concentration of flavonoids and total phenols in lettuce is sensitive to environmental conditions (Liu et al., 2007). Under high light stress, polyphenols are often produced in plant tissues as some form of protective agent (Chalker-Scott, 1999). Anthocyanins, as one of the most abundant pigments in higher plants, increase with the increase of light intensity, which helps to improve the coloring ability and antioxidant capacity of leaves, while protecting plants from photodamage and photoinhibition in the face of excessive solar radiation (Gitelson et al., 2007; Zhang et al., 2019). In this study, the anthocyanins, total phenols and flavonoids contents of celery leaves and petioles increased with the increase of light intensity, and the activity of phenylalanine ammonia-lyase (PAL), as a key enzyme in the synthesis of phenols, increased under the induction of strong light (**Figures 4A, C**). Studies have shown that flavonoids contain many chemicals (Williams and Grayer, 2004). During the S3 period, there is a sudden increase in the total flavonoid content in the leaves, possibly the accumulation of other flavonoids.

The flavonoid content in Cabernet Sauvignon grapes increased significantly under high light intensity conditions (Koyama et al., 2012). PAL, CHS, CHI, FNS and ANS are the more critical enzymes for the synthesis of apigenins and anthocyanins in plants (Yan et al., 2020; Dai et al., 2022). The expression of related genes in the metabolic pathway of anthocyanin pre-synthesis is regulated by light intensity (Zhang et al., 2018). It was found that the expression of structural and regulatory genes in the anthocyanin synthesis and metabolism pathway was up-regulated in plants with increased anthocyanin content under high light conditions, while in low light or dark conditions, anthocyanin-related genes were not expressed or their expression was down-regulated leading to a significant decrease in anthocyanin (An et al., 2023). Similar to the results of the present study, PAL, CHS and CHI activities in celery increased and then decreased with increasing light intensity in a certain light intensity range under different light intensity conditions (**Figure 5A–C**). This indicates that the activity of these three enzymes is different in different light intensity treatments and in different parts of the plant, which is similar to the previous findings, and its activity increases with the increase of light intensity (Dong et al., 1998; Qiu, 2016).

The results showed that light intensity was the key factor affecting the synthesis of anthocyanins in tartary buckwheat, and the content of anthocyanins changed significantly (Wang D. et al., 2022). In this study, the content of anthocyanins in leaves and petioles increased with the increase of light intensity at 15, 30 and 45 days, and the activity of ANS enzyme and the expression of the key gene *Ag3GT* gene were also up-regulated during the synthesis of anthocyanins under strong light (**Figures 5D**, **6A**). Besides, we found that the activity of FNS, a key enzyme for apigenin synthesis, and the expression of *AgFNS*, a key gene, were consistently decreased with increasing treatment time under the same light treatment, which was opposite to the expression trend of ANS, a key enzyme for anthocyanin synthesis, and *Ag3GT*, a key gene (**Figures 5D, E**, **6**). This is consistent with the previous research on the competition between anthocyanins and apigenin synthesis in celery (Martens and Forkmann, 1998; Gebhardt et al., 2005).

## 5 Conclusion

The light intensity treatment of 200 μmol·m^-2^·s^-1^ significantly improved the yield and quality of celery, and also reduced the nitrate content. The content of nitrate and cellulose in celery leaves is lower than that of petioles, and the content of other nutrients in leaves is higher than that of petioles. The contents of apigenin and anthocyanins were higher at 200 μmol·m^-2^·s^-1^, and higher at 30 d. The light intensity of 300 μmol·m^-2^·s^-1^ inhibited the activities of CHS, CHI and FNS. The expression of *AgFNS*, a key gene for apigenin synthesis, was up-regulated with the increase of light intensity, and was down-regulated at 300 μmol·m^-2^·s^-1^, indicating that the expression of *Ag3GT*, a key gene for anthocyanin synthesis in celery, was proportional to the light intensity. Correlation analysis showed that 200 μmol·m^-2^·s^-1^ was the optimal light intensity for celery growth.

## Data availability statement

The original contributions presented in the study are included in the article/supplementary material. Further inquiries can be directed to the corresponding author.

## Author contributions

ZH: Conceptualization, Methodology, Project administration, Writing – review & editing. YQ: Formal analysis, Writing – original draft, Writing – review & editing. XL: Formal analysis, Writing – original draft, Writing – review & editing. CL: Writing – review & editing. SC: Writing – review & editing. QC: Writing – review & editing. SL: Writing – review & editing. DS: Investigation, Writing – review & editing. XG: Investigation, Writing – review & editing. XZ: Writing – review & editing. LS: Methodology, Writing – review & editing.
